# The Relationship Between Daily Dietary Intake of Fiber and Short Sleep Duration in the Presence of Di(2-Ethylhexyl) Phthalate: A Population-Based Study

**DOI:** 10.3389/fnut.2022.910892

**Published:** 2022-06-15

**Authors:** Jilei Lin, Siying Cheng, Jing Zhang, Shuhua Yuan, Lei Zhang, Jinhong Wu, Jiande Chen, Mingyu Tang, Liebin Zhao, Yong Yin

**Affiliations:** ^1^Department of Respiratory Medicine, Shanghai Children's Medical Center, School of Medicine, Shanghai Jiao Tong University, Shanghai, China; ^2^Department of Neurology, Renji Hospital, School of Medicine, Shanghai Jiao Tong University, Shanghai, China; ^3^Shanghai Engineering Research Center of Intelligence Pediatrics, Shanghai, China; ^4^Pediatric AI Clinical Application and Research Center, Shanghai Children's Medical Center, Shanghai, China

**Keywords:** di(2-ethylhexyl) phthalate, short sleep duration, daily dietary intake of fiber, dose-response analysis, National Health and Nutrition Examination Survey

## Abstract

**Objective:**

This study aimed to evaluate the relationship between daily dietary intake of fiber (DDIF) and short sleep duration (SSD) in the presence of di(2-ethylhexyl) phthalate.

**Methods:**

Data of 13,634 participants in this study were collected from the National Health and Nutrition Examination Survey (NHANES). The sum of urinary mono-2-ethyl-5-carboxypentyl phthalate, mono-(2-ethyl-5-hydroxyhexyl) phthalate, mono-(2-ethyl)-hexyl phthalate, and mono-(2-ethyl-5-oxohexyl) phthalate was used to evaluate the level of di(2-ethylhexyl) phthalate (DEHP) exposure. The ln-transformed urinary creatinine-corrected DEHP [ln(DEHP/UCr)] level was used in the statistical models. DDIF was divided into tertiles (<5.77 g/1,000 kcal, 5.77–9.04 g/1,000 kcal, and ≥9.04 g/1,000 kcal).

**Results:**

The 13,634 participants included in this study were classified into two groups according to sleep duration. The dose response analysis showed that higher ln(DEHP/UCr) was related to a higher risk of SSD (<7 h and <6 h). Participants in the highest vs. the lowest quartile of DEHP were found to be at increased risk of SSD (<7 h, <6 h, and <5 h). The result of risk of SSD <7 h was OR 1.57, 95% CI, 1.40–1.76; P_trend_ <0.001, of SSD <6 h was OR 1.38, 95% CI, 1.18–1.61; P_trend_ <0.001, and of SSD <5 h was OR 1.45, 95% CI, 1.13–1.86; P_trend_ <0.001. DEHP exposure was found to be associated with SSD <7 h in a sex-specific manner (P_interaction_ = 0.033). A significant interaction between ln(DEHP/UCr) and DDIF_(tertiles1 vs. tertiles2)_ (P_interaction_ = 0.02) was detected for SSD <7 h.

**Conclusion:**

Our results showed that there was a harmful association between DEHP exposure and SSD (<7 h, <6 h, and <5 h). The ameliorative effects of median level of DDIF on SSD <7 h in the presence of DEHP exposure were observed in this study.

## Introduction

Di(2-ethylhexyl) phthalate (DEHP), one of the phthalates family, is used as the most common plasticizer in many products, including medical tubes and bags, food, and beverage packaging (1). Due to the high exposure to DEHP in daily life, eight of its metabolites have been detected in human biospecimens (2). The concentration of DEHP metabolites in urine was the most commonly used way to assess the level of DEHP exposure, because urine excretes DEHP metabolites (3). As a kind of endocrine disruptor, it was reported that DEHP exposure was related to endocrine, reproductive, and neural diseases (4–6). DEHP was thought to be associated with sleep disorder by changing the levels of reproductive hormones (7, 8). One study suggested that the higher concentration of DEHP in urine and higher levels of estradiol: progesterone were observed in women with insomnia (9). Sleep is essential for mental health and wellbeing. However, a large number of people have sleep difficulties and suffer from short sleep duration (SSD) (10). In the United States, approximately 29.2% of adults do not get the recommended duration of sleep (11). However, there is no research with a large sample size to comprehensively explore the effects of DEHP exposure on sleep duration in the US population.

Dietary fiber is an essential nutrient, found in fruits, vegetables, and whole grains. It is widely recognized that dietary fiber intake helps with the reduction of diseases (12). Previous studies showed that Americans with normal sleep duration have the highest level of daily dietary intake of fiber (DDIF) (13). However, the role of DDIF on the effects of SSD in the presence of DEHP is unknown.

Considering the need for data from large-population studies on this topic in the US population, we performed a population-based study to evaluate the relationship between DEHP exposure and SSD. Meanwhile, this study also explored the role of DDIF on SSD in the presence of DEHP exposure in the US population.

## Methods

### Study Population

In this study, we used publicly available data from the National Health and Nutrition Examination Survey (NHANES), which is a national population-based survey designed to collect information on the health and nutrition status of the US population conducted by the CDC (14). Seven consecutive NHANES cycles (2005–2006, 2007–2008, 2009–2010, 2011–2012, 2013–2014, 2015–2016, 2017–2018) from the NHANES database were included in this study. Data on dietary fiber intake, alternatives of phthalates, and sleep duration were retrieved from the database. Finally, 13,634 participants were included in our study.

### Data Extraction

Demographic information [age (years old), gender (man, woman), body mass index (BMI, kg/m2), race (other Hispanic, non-Hispanic White, non-Hispanic Black, other race, Mexican American), level of education (below high school, high school, above high school)], underlying disease (history of hypertension, history of diabetes), concentration of DEHP in urine (see the following definition), sleep duration, and DDIF were included in this study. We excluded those participants who did not complete the questionnaire on sleep, who did not undergo urinary DEHP detection, and of whom the results of urinary DEHP detection or the results of urinary creatinine were missing. We used the is.na function in R software to verify the integrity of the data.

### Exposure of DEHP

In this study, four kinds of DEHP metabolites in urine were detected during the seven consecutive NHANES cycles: mono-2-ethyl-5-carboxypentyl phthalate (MECPP), mono-(2-ethyl-5-hydroxyhexyl) phthalate (MEHHP), mono-(2-ethyl)-hexyl phthalate (MEHP), mono-(2-ethyl-5-oxohexyl) phthalate (MEOHP). For the values of these metabolites below the lower limit of detection (LLOD), the values would be divided by the square root of 2 [LLOD/sqrt (2)]. The level of DEHP exposure was calculated as the sum of MECPP, MEHHP, MEHP, and MEOHP in this study. The metabolite concentrations were adjusted using urinary creatinine (UCr) levels. ln-transformed creatinine-adjusted DEHP [ln(DEHP/UCr)] was used in all analyses to convert the data distribution into normal distribution.

### Short Sleep Duration

Sleep duration in the NHANES was measured by a self-report questionnaire: “How much sleep do you usually get at night on weekdays or workdays?” In five consecutive NHANES cycles (2005–2006, 2007–2008, 2009–2010, 2011–2012, 2013–2014), participants reported the hours of sleep duration with the integers ranging from 2 to 12 (>12 h is considered as 12 h). In two consecutive NHANES cycles (2015–2016, 2017–2018), participants reported the hours of sleep duration ranging from 2 to 14 (>14 h is considered as 14 h). We classified sleep time into three kinds of SSD (<7 h, <6 h, or <5 h).

### Daily Dietary Intake of Fiber

The 24-h detailed dietary intake information was collected in the dietary interview. The US Department of Agriculture's Food and Nutrient Database for Dietary Studies (FNDDS) was used for evaluating the nutrient intakes (http://www.ars.usda.gov/ba/bhnrc/fsrg). The FNDDS includes comprehensive information that can be used to code individual foods reported by participants and also includes nutrient values for calculating nutrient intakes. Foods/beverages or portions that could not be matched to items in the database were resolved by scientists. Finally, DDIF in grams (g) was calculated from the total food and beverages consumption, and DDIF was adjusted by total dietary energy (kcal) levels. The DDIF of 90% of the population was lower than the recommended intake of daily fiber intake (14 g/1,000 kcal) for healthy adults; therefore, we divided DDIR into three groups by its tertiles (T1: <5.77 g/1,000 kcal, T2: 5.77–9.04 g/1,000 kcal, T3: ≥9.04 g/1,000 kcal).

### Statistical Analysis

Baseline variables were presented as the mean ± SD. Participants were classified into four groups according to quartiles of ln(DEHP/UCr). Logistic regression was applied to estimate odds ratio (ORs) and 95% confidence intervals (CIs) for the association between the level of DEHP exposure and the risk of SSD. The model was adjusted by gender (man or woman), age (years old), race (Mexican American, other Hispanic, non-Hispanic White, non-Hispanic Black, and other race), education (below high school, high school, above high school), history of diabetes (yes or no), and hypertension (yes or no). Prespecified subgroup analyses were conducted to assess whether the observed association of level of DEHP exposure and the risk of SSD was modified by gender (man or woman), age (years old), race (other Hispanic, non-Hispanic White, non-Hispanic Black, other race, Mexican American), education (high school, above high school, below high school), history of diabetes, and history of hypertension. A P_interaction_ was obtained through a likelihood ratio test, which compares the models with and without interaction terms. Restricted cubic spline regression with three knots at the 10th, 50th, and 90th percentiles was used to explore the potential dose–response relationship between DEHP and SSD (15). The reference level was set at 5.03 (ng/L)/(g/dL). A P_nonlinearity_ was obtained by testing the null hypothesis that regression coefficients of the second splines are equal to zero. A value of *P* < *0*.05 was considered to indicate statistical significance. All statistical analyses were performed using SPSS 25.0 software and R 4.1 software.

## Results

### Characteristics of the Included Population

In general, 70,190 subjects were invited to participate in the NHANES cross-sectional study between 2005–2018. Participants without results of DEHP metabolites and sleep duration were excluded; finally, 13,634 participants were included in this study. The detailed information of the screening process is shown in Supplementary Figure S1. The mean age of all participants was 45.34 ± 20.32 years old. A total of 9,005 cases (4,365 men and 4,640 women) were divided into group 1 (sleep duration ≥7 h) and 4,629 cases (2,345 men and 2,284 women) were divided into group 2 (sleep duration <7 h) (Table 1). There were significant differences in BMI, race, education, history of diabetes, and history of hypertension between the two groups. The four kinds of DEHP metabolites (MECPP, MEHHP, MEHP, MEOHP) and their sum were significantly different between the two groups with all of them higher in the group of SSD <7 h.

**Table 1:** The characteristics and concentration of urinary DEHP metabolites in the included population.

	**Total**	**Sleep duration**		
		**≥7 h**	** <7 h**	***P***
**Sample size**	13,634	9,005	4,629	
**Gender (men, %)**	6,710 (49.22%)	4,365 (48.47%)	2,345 (50.66%)	0.016
**Age (m** **±sd, years old)**	45.58 ± 19.59	45.34 ±20.32	46.06 ±18.08	0.034
**BMI (m** **±sd, kg/m2)**	28.78 ±7.07	28.4 ±6.91	29.52 ±7.31	<0.001
**Race**				<0.001
Mexican American	2,237 (16.41%)	1,583 (17.58%)	654 (14.13%)	
Other Hispanic	1,281 (9.4%)	820 (9.11%)	461 (9.96%)	
Non-Hispanic white	5,443 (39.92%)	3,816 (42.38%)	1,627 (35.15%)	
Non-Hispanic black	3,089 (22.66%)	1,700 (18.88%)	1,389 (30.01%)	
Other race	1,584 (11.62%)	1,086 (12.06%)	498 (10.76%)	
**Education**				0.009
Below high school	4,084 (29.99%)	1,954 (25.08%)	1,063 (24.9%)	
High school	3,074 (22.57%)	1,736 (22.28%)	1,053 (24.67%)	
Above high school	6,462 (47.44%)	4,101 (52.64%)	2,153 (50.43%)	
**History of diabetes**	1,544 (11.33%)	967 (10.75%)	577 (12.46%)	0.003
**History of hypertension**	4,358 (31.99%)	2,721 (30.25%)	1,637 (35.39%)	<0.001
**The ln-transformed of [DEHP (ng/L)/ urinary creatinine (g/dL)]**				
MECPP	7.33 ± 1.07	7.29 ± 1.05	7.4 ± 1.08	<0.001
MEHHP	6.88 ± 1.13	6.82 ± 1.12	6.98 ± 1.15	<0.001
MEHP	5.1 ± 1.09	5.08 ± 1.07	5.15 ± 1.12	0.001
MEOHP	6.41 ± 1.09	6.36 ± 1.08	6.5 ± 1.1	<0.001
DEHP	8.13 ± 1.05	8.08 ± 1.04	8.21 ± 1.07	<0.001
**Daily dietary intake of fiber (g/1,000 kcal)**	8.17 ± 4.55	8.38 ± 4.63	7.78 ± 4.36	<0.001
**Short sleep duration**				
<7 h	4,629 (33.95%)	0(0%)	4,629 (100%)	-
<6 h	1,798 (13.19%)	0(0%)	1,798 (13.19%)	-
<5 h	646 (4.74%)	0(0%)	646 (4.74%)	-

*BMI, body mass index; DEHP, di-2-ethylhexyl phthalate, DEHP=MECPP+MEHHP+ MEHP+MEHP; MECPP, mono-2-ethyl-5-carboxypentyl phthalate, MEHHP, mono-(2-ethyl-5-hydroxyhexyl) phthalate, MEHP: mono-(2-ethyl)-hexyl phthalate, MEOHP, mono-(2-ethyl-5-oxohexyl) phthalate; m ± sd, mean ± standard deviation*.

### Subjects With Different DEHP Exposure

All participants were divided by quartiles of ln(DEHP/UCr). The clinical characteristics of the four groups are shown in Table 2. Participants in the highest vs. the lowest quartiles of DEHP were older and more likely to be women, non-Hispanic White, or Mexican American, more likely to have a lower education, a history of hypertension and diabetes, and more likely to suffer from SSD (<7 h, <6 h, and <5 h) (all P_trend_<*0*.05) (Table 2).

**Table 2:** The clinical characteristics of the study population according to the concentration of DHEP metabolites in urine.

**Characteristics**	**Quartile of ln(DEHP/UCr), range (median)**				**P_**trend**_**
	**5.03**~**7.42 (7.05)**	**7.42**~**8.00 (7.73)**	**8.00**~**8.67 (8.29)**	**8.67**~**14.55 (9.25)**	
**Sample size**	3,431	3,409	3,407	3,387	
**Gender [men, %]**	1,965 (57.27%)	1,726 (50.63%)	1,580 (46.38%)	1,439 (42.49%)	<0.001
**Age (m** **±sd, years old)**	43.11 ± 18.84	45.73 ± 19.58	47.58 ± 19.62	45.95 ± 20.04	<0.001
**BMI (m** **±sd, kg/m2)**	28.76 ± 7.38	28.81 ± 7.01	28.9 ± 7.00	28.65 ± 6.87	0.679
**Race**					<0.001
Mexican American	442 (12.88%)	521 (15.28%)	595 (17.46%)	679 (20.05%)	
Other Hispanic	309 (9.01%)	316 (9.27%)	328 (9.63%)	328 (9.68%)	
Non-Hispanic White	1,184 (34.51%)	1,405 (41.21%)	1,388 (40.74%)	1,466 (43.28%)	
Non-Hispanic Black	968 (28.21%)	763 (22.38%)	727 (21.34%)	631 (18.63%)	
Other race	528 (15.39%)	404 (11.85%)	369 (10.83%)	283 (8.36%)	
**Education**					<0.001
Below high school	593 (19.87%)	730 (24.11%)	848 (27.53%)	846 (28.50%)	
High school	701 (23.49%)	713 (23.55%)	688 (22.34%)	687 (23.15%)	
Above high school	1,690 (56.64%)	1,585 (52.34%)	1,544 (50.13%)	1,435 (48.35%)	
**History of diabetes**	302 (8.81%)	396 (11.62%)	410 (12.04%)	436 (12.88%)	<0.001
**History of hypertension**	1,028 (29.98%)	1,101 (32.33%)	1,151 (33.81%)	1,078 (31.86%)	0.045
**Sleep duration**					
<7 h	1,025 (29.87%)	1,144 (33.56%)	1,185 (34.78%)	1,275 (37.64%)	<0.001
<6 h	408 (11.89%)	428 (12.56%)	474 (13.91%)	488 (14.41%)	0.001
<5 h	137 (3.99%)	163 (4.78%)	170 (4.99%)	176 (5.20%)	0.019

*DEHP, di-2-ethylhexyl phthalate, DEHP = MECPP+ MEHHP+ MEHP+ MEOHP; ln(DEHP/UCr), ln[di-2-ethylhexyl phthalate(ng/L)/urinary creatinine(g/dL)]; BMI, body mass index; m ± sd, mean ± standard deviation*.

### Effects of DEHP Exposure on Short Sleep Duration

**Figure 1:**
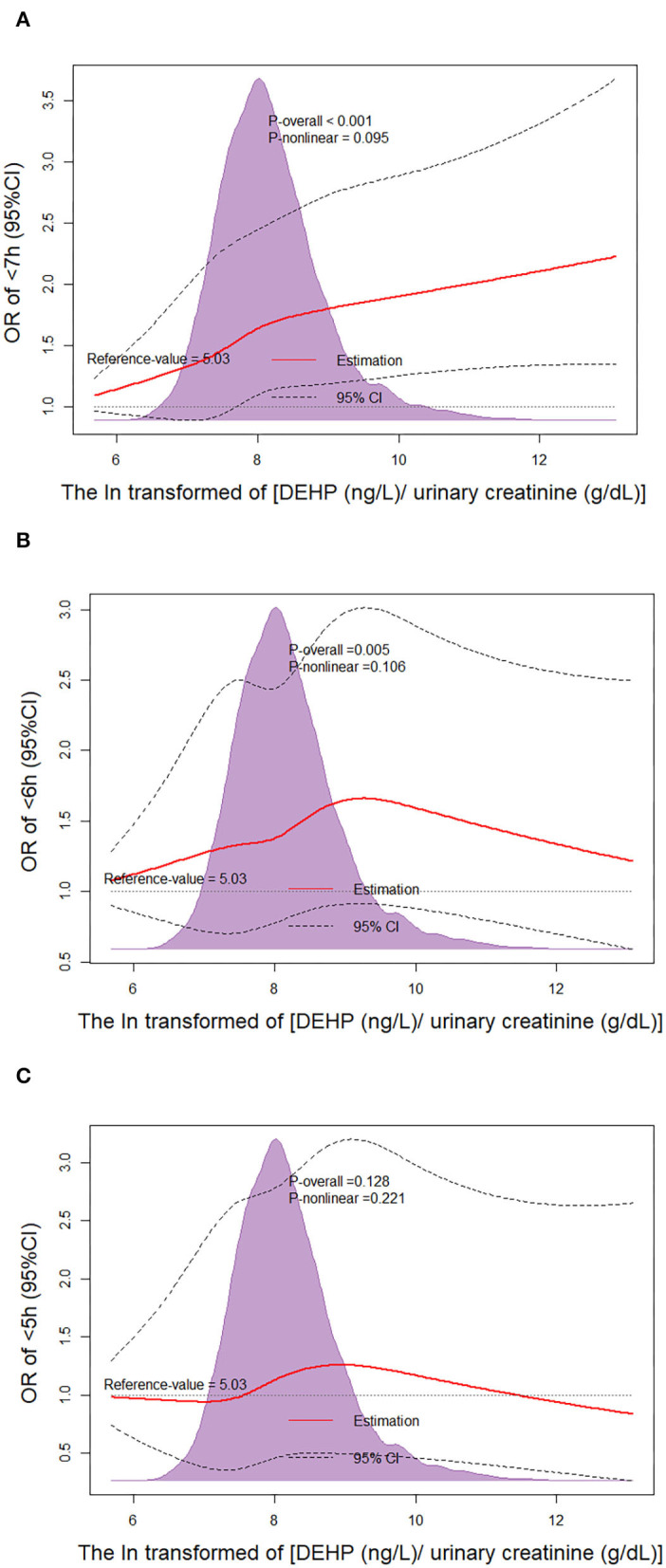
Non-linear dose–response analysis on urinary di(2-ethylhexyl) phthalate (DEHP) concentration and short sleep duration (SSD). Restricted cubic spline regression with three knots at the 10th, 50th, and 90th percentiles was used to explore the potential dose–response relationship between DEHP and SSD. The reference level was set at 5.03. A P_non−linearity_ was obtained by testing the null hypothesis that the regression coefficient of the second spline was equal to zero. CI, confidence interval; **(A)** Non-linear dose–response analysis on ln(DEHP/UCr) and SSD <7 h; **(B)** Non-linear dose–response analysis on ln(DEHP/UCr) and SSD <6 h; **(C)** Non-linear dose–response analysis on ln(DEHP/UCr) and SSD <5 h.

We performed dose-response analysis of the relationship between ln(DEHP/UCr) and SSD. It was observed that the higher urinary DEHP was related with a higher risk of SSD (<7 h and <6 h) with *P-*values.001 and.005, respectively. However, the *P-*value for the non-linear dose–response manner between ln(DEHP/UCr) and SSD (<7 h and <6 h) was insignificant (P_nonlinearity_ = 0.095 and.106, respectively) (Figure 1).

The associations of DEHP and SSD among the four groups are shown in Table 3. Participants in the highest quartile of ln(DEHP/UCr) were associated with higher risks of SSD compared with participants in the lowest quartile. After the full adjustment for confounders (gender, age, race, education, history of diabetes and hypertension), the result of risk in SSD <7 h was OR _quartiles4_ _vs.1_ 1.59, 95% CI, 1.43–1.76; P_trend_<*0*.001, in SSD <6 h was OR _quartiles4_ _vs.1_ 1.39, 95% CI, 1.208–1.60; P_trend_<*0*.001, and in SSD <5 h was OR _quartiles4_ _vs.1_ 1.42, 95% CI, 1.13–1.80; P_trend_<*0*.001 (Table 3).

**Table 3:** Association between DEHP exposure and SSD.

**Outcomes**	**Model**	**Quartile of ln(DEHP/UCr), range (median)**				***P*_trend_**
		**5.03**~**7.42 (7.05)**	**7.42**~**8.00 (7.73)**	**8.00**~**8.67 (8.29)**	**8.67**~**14.55 (9.25)**	
SSD <7 h	Model 1	1.00 (Reference)	1.19 (1.07, 1.31)	1.25 (1.13, 1.39)	1.42 (1.28, 1.57)	<0.001
	Model 2	1.00 (Reference)	1.26 (1.13, 1.40)	1.35 (1.22, 1.50)	1.59 (1.43, 1.76)	<0.001
	Model 3	1.00 (Reference)	1.26 (1.13, 1.40)	1.35 (1.21, 1.50)	1.60 (1.44, 1.78)	<0.001
	Model 4	1.00 (Reference)	1.26 (1.13, 1.39)	1.35 (1.22, 1.50)	1.59 (1.43, 1.76)	<0.001
SSD <6 h	Model 1	1.00 (Reference)	1.06 (0.92, 1.23)	1.20 (1.04, 1.38)	1.25 (1.08, 1.44)	0.001
	Model 2	1.00 (Reference)	1.13 (0.97, 1.30)	1.28 (1.11, 1.48)	1.39 (1.20, 1.61)	<0.001
	Model 3	1.00 (Reference)	1.12 (0.96, 1.29)	1.28 (1.10, 1.48)	1.39 (1.20, 1.61)	<0.001
	Model 4	1.00 (Reference)	1.13 (0.97, 1.30)	1.28 (1.11, 1.48)	1.39 (1.20, 1.60)	<0.001
SSD <5 h	Model 1	1.00 (Reference)	1.21 (0.96, 1.52)	1.26 (1.00, 1.59)	1.32 (1.05, 1.66)	0.019
	Model 2	1.00 (Reference)	1.25 (0.99, 1.58)	1.31 (1.04, 1.66)	1.43 (1.13, 1.81)	0.003
	Model 3	1.00 (Reference)	1.23 (0.97, 1.56)	1.31 (1.04, 1.66)	1.43 (1.13, 1.81)	0.003
	Model 4	1.00 (Reference)	1.25 (0.99, 1.58)	1.31 (1.04, 1.66)	1.42 (1.13, 1.80)	0.007

*SSD, shorter sleep duration; DEHP, di-2-ethylhexyl phthalate, ln(DEHP/UCr), ln[di-2-ethylhexyl phthalate(ng/L)/urinary creatinine(g/dL)]; BMI, body mass index.*

*Model 1: Unadjusted model.*

*Model 2: Adjusted for gender (man or woman), age (years old), race (Mexican American, other Hispanic, non-Hispanic White, non-Hispanic Black, and other race), education (below high school, high school, above high school).*

*Model 3: Adjusted for gender (man or woman), age (years old), race (Mexican American, other Hispanic, non-Hispanic White, non-Hispanic Black, and other race), education (below high school, high school, above high school), BMI (kg/m2), history of diabetes (yes or no), and hypertension (yes or no).*

*Model 4: Adjusted for gender (man or woman), age (years old), race (Mexican American, other Hispanic, non-Hispanic White, non-Hispanic Black, and other race), education (below high school, high school, above high school), history of diabetes (yes or no), and hypertension (yes or no)*.

### Subgroup Analyses

The subgroup analyses on the associations of ln(DEHP/UCr) (highest vs. lowest quartiles of DEHP) and all of SSD are shown in Table 4 and Supplementary Tables S1, S2. Significant interactions were detected between DEHP and DDIF (T2 vs. T1) or sex (woman vs. man) for SSD <7 h (P_interaction_ = 0.020 and 0.033, respectively), but not for SSD <6 h and <5 h. Specifically, women was found to be associated with higher risks of SSD <7 h in the presence of DEHP (OR _quartiles4vs.1_, 1.78; 95% CI, 1.50–2.10) than men (OR _quartiles4vs.1_, 1.39; 95% CI, 1.68–1.63), with a P_trend_ of 0.033. T1 of DDIF was found to be associated with higher risks of SSD <7 h in the presence of DEHP (OR _quartiles4vs.1_, 1.82; 95% CI, 1.52–2.19) than T2 of DDIF (OR _quartiles4vs.1_, 1.47; 95% CI, 1.21–1.79), with a P_trend_ of 0.02. However, no significant interaction was found for the remaining predefined factors.

**Table 4:** Subgroup analyses on the association between DEHP in urine and SSD <7 h.

**Subgroup variable**	**Total number** **=** **6,383, SSD** ** <7 h** **=** **2,172**		
	**OR_**quartile 4 vs. 1**_ (95% CI)**	***P***	***P*_interaction_**
**Gender**			
Woman	1.78 (1.50, 2.10)	<0.001	
Man	1.39 (1.18, 1.63)	<0.001	0.033
**Age**			
<60	1.60 (1.40, 1.84)	<0.001	
≥60	1.37 (1.11, 1.69)	0.004	0.166
**Race**			
Mexican American	1.41 (1.04, 1.93)	0.028	
Other Hispanic	1.16 (0.81, 1.68)	0.138	0.542
Non-Hispanic White	1.91 (1.58, 2.32)	<0.001	0.126
Non-Hispanic Black	1.49 (1.18, 1.87)	<0.001	0.737
Other race	1.47 (1.05, 2.07)	0.025	0.780
**Education**			
Below high school	1.46 (1.15, 1.86)	0.002	
High school	1.93 (1.53, 2.46)	<0.001	0.063
Above high school	1.46 (1.25, 1.72)	<0.001	0.703
**History of diabetes**			
No	1.58 (1.40, 1.79)	<0.001	
Yes	1.41 (1.02, 1.98)	0.042	0.583
**History of hypertension**			
No	1.63 (1.41, 1.88)	<0.001	
Yes	1.47 (1.21, 1.79)	0.016	0.647
**DDIF (g/1,000 kcal)**			
T1	1.82 (1.52, 2.19)	<0.001	
T2	1.47 (1.21, 1.79)	<0.001	0.020
T3	1.49 (1.22, 1.82)	<0.001	0.060

*DEHP=MECPP+MEHHP+ MEHP+MEHP; the values of DEHP: urinary DEHP (ng/ml)/ urinary creatinine (mg/ml); SSD, short sleep duration; DDIF, daily dietary intake of fiber (g/1,000 kcal), T1: <5.77 g/1,000 kcal, T2: 5.77–9.04 g/1,000 kcal, T3: ≥9.04 g/1,000 kcal; OR is adjusted for gender (man or woman), age (years old), race (Mexican American, other Hispanic, non-Hispanic White, non-Hispanic Black, and other race), education (below high school, high school, above high school), history of diabetes (yes or no), and hypertension (yes or no)*.

### Appropriate Level of DDIF Protects Against the SSD in the Presence of DEHP

A total of 885 participants were excluded due to missing data of DDIF in this analysis. First of all, 12,749 participants were divided into three groups according to the tertiles of DDIF (T1, T2, and T3); furthermore, participants in each group were divided into four groups according to the quartiles of ln(DEHP/UCr). Detailed information is shown in Table 5 and Supplementary Tables S3, S4. In general, after the full adjustment for confounders, participants in the higher ln(DEHP/UCr) groups were still associated with higher risks of SSD (<7 h and <6 h) in each group of DDIF (T1, T2, and T3) (all P_trend_<*0*.05) (Table 5 and Supplementary Table S3). Participants in T2 or T3 of DDIF were associated with lower risks of SSD (<7 h and <6 h) in the presence of DEHP compared with T1. However, the risks of SSD <7 h in T2 and T3 groups of DDIF were still significantly higher in the highest quartile of ln(DEHP/UCr) compared with the lowest quartile of ln(DEHP/UCr).

**Table 5:** Association between DEHP exposure and short sleep duration <7 h in different levels of DDIF.

	**DIDF**	***N***	**Model**	**Quartile of ln(DEHP/UCr), range (median)**				***P*_trend_**
				**5.03**~**7.42 (7.05)**	**7.42**~**8.00 (7.73)**	**8.00**~**8.67 (8.29)**	**8.67**~**14.55 (9.25)**	
SSD <7 h	T1	4,246	Model 1	1.00 (Reference)	1.43 (1.20, 1.71)	1.39 (1.16, 1.66)	1.72 (1.44, 2.05)	<0.001
			Model 2	1.00 (Reference)	1.49 (1.24, 1.79)	1.45 (1.21, 1.74)	1.82 (1.52, 2.17)	<0.001
			Model 3	1.00 (Reference)	1.48 (1.23, 1.77)	1.44 (1.20, 1.73)	1.81 (1.52, 2.17)	<0.001
			Model 4	1.00 (Reference)	1.47 (1.23, 1.77)	1.44 (1.20, 1.72)	1.81 (1.51, 2.17)	<0.001
	T2	4,260	Model 1	1.00 (Reference)	1.16 (0.97, 1.39)	1.29 (1.08, 1.54)	1.23 (1.03, 1.47)	0.013
			Model 2	1.00 (Reference)	1.26 (1.05, 1.52)	1.42 (1.18, 1.71)	1.44 (1.20, 1.74)	<0.001
			Model 3	1.00 (Reference)	1.26 (1.04, 1.51)	1.41 (1.17, 1.70)	1.47 (1.21, 1.77)	<0.001
			Model 4	1.00 (Reference)	1.27 (1.06, 1.53)	1.43 (1.19, 1.72)	1.45 (1.20, 1.75)	<0.001
	T3	4,243	Model 1	1.00 (Reference)	0.99 (0.82, 1.20)	1.09 (0.90, 1.32)	1.34 (1.12, 1.62)	<0.001
			Model 2	1.00 (Reference)	1.03 (0.85, 1.26)	1.18 (0.97, 1.43)	1.48 (1.22, 1.80)	<0.001
			Model 3	1.00 (Reference)	1.05 (0.86, 1.27)	1.19 (0.97, 1.44)	1.50 (1.24, 1.83)	<0.001
			Model 4	1.00 (Reference)	1.04 (0.86, 1.26)	1.19 (0.98, 1.44)	1.47 (1.22, 1.79)	<0.001

*DEHP=MECPP+MEHHP+ MEHP+MEHP; the values of DEHP: urinary DEHP (ng/L)/ urinary creatinine (g/dL); SSD, short sleep duration; DDIF, daily dietary intake of fiber (g/1,000 kcal).*

*T1: <5.77 g/1,000 kcal, T2: 5.77–9.04 g/1,000 kcal, T3: ≥9.04 g/1,000 kcal.*

*Model 1: Unadjusted model.*

*Model 2: Adjusted for gender (man or woman), age (years old), race (Mexican American, other Hispanic, non-Hispanic White, non-Hispanic Black, and other race), education (below high school, high school, above high school).*

*Model 3: Adjusted for gender (man or woman), age (years old), race (Mexican American, other Hispanic, non-Hispanic White, non-Hispanic Black, and other race), education (below high school, high school, above high school), BMI (kg/m2), history of diabetes (yes or no), and hypertension (yes or no).*

*Model 4: Adjusted for gender (man or woman), age (years old), race (Mexican American, other Hispanic, non-Hispanic White, non-Hispanic Black, and other race), education (below high school, high school, above high school), history of diabetes (yes or no), and hypertension (yes or no)*.

## Discussion

In this large cross-sectional study, we revealed significant associations of DEHP exposure and the risk of SSD. The higher concentration of DEHP metabolites in urine was found to be associated with a higher risk of SSD (<7 h, <6 h, and <5 h). Subgroup analyses further showed that the harmful association of DEHP and SSD <7 h were more pronounced in the lowest DDIF group than in the median DDIF group.

Although, a large number of studies have shown that DEHP exposure is harmful for human health, there are few studies on the effect of DEHP on sleep. Our results showed that the level of DEHP in urine was related with the risk of SSD in the whole population. Compared with one previous study with only a small sample of women (9), our study showed that DEHP affected a wide range of the US population. The results of our subgroup analyses showed a significant interaction between DEHP and gender (man vs. woman), which indicated that the results of studies with only one gender need to be interpreted carefully. The opposite outcomes of SSD <5 h of participants in DEHPgroups might be related to the small sample size; therefore, the results of studies with limited sample size on this topic should be interpreted carefully.

In this study, the results of our dose-response analysis suggested that the increasing ln(DEHP/UCr) significantly increased the risk of SSD (<7 h and <6 h) without a non-linear dose–response trend. However, the urinary concentration of DEHP was ln-transformed in analysis, and the true relationship between urinary DEHP and risk of SSD (<7 h and <6 h) is likely to be a dose relationship. Note that Figure 1 shows that the trend of OR in the group of <6 h and the group of <5 h is different from the group of <7 h. Previous studies showed that people with more severe SSD were related with higher risk of cardiovascular diseases, depression, and abuse of tobacco and alcohol products (16, 17). These factors may mask the effect of DEHP on SSD. However, the kind of relationship that exists between DEHP and SSD is insignificant because the higher DEHP should be related to a higher risk of SSD (<7 h and <6 h). At present, few studies focused on the mechanism of SSD occurrence associated with DEHP exposure. Previous studies showed that an imbalance of reproductive hormones may contribute to poor sleep duration (18, 19). DEHP is a class of environmentally pervasive endocrine disruptors to humans and mice, and it was reported that DEHP exposure was related to increased serum estradiol concentrations and decreased serum testosterone and follicle stimulating hormone (20). The reduction of progesterone secretion caused by DEHP exposure might lead to SSD; therefore, the sleep quality of women may be more likely to be affected by DEHP exposure. This is similar to the results of our subgroup analysis. However, higher DEHP exposure was also related to a significantly higher risk of SSD (<7 h and <6 h) of men in our subgroup analysis, which indicated that the mechanism will be more complex.

Interestingly, our subgroup analysis suggested that the risk of SSD <7 h of participants in tertile 2 of DDIF were less likely to be affected by DEHP than patients in tertile 1 of DDIF. The interaction between dietary fiber intake and phthalates exposure is still unknown. Some previous studies suggested that high DDIF was negatively associated with sleep trouble (21, 22). One previous study showed that high DDIF could regulate the hormone concentrations in women (23). Therefore, the regulation of hormones might be the underlying mechanism for the interaction between DDIF and DEHP exposure affecting SSD.

Our results showed that social determinants of health such as gender and race were related to the level of phthalate exposure. Similar results were shown in previous studies that men and non-Hispanic Black participants were related to lower levels of phthalate exposure (24–26). Food and beverages were usually held in a plastic container, and different eating habits may lead to the difference in phthalate exposure levels. It might be the reason why the non-Hispanic black population has lower levels of phthalate exposure. The fact that women use more plastic decorations and cosmetics containing PAE, such as nail polish may contribute to the higher level of phthalate exposure in women.

There were several strengths in this study. First, the sample size of our study is considerably large, which contributes to the stability of the result. Second, not only did we find a high correlation between DEHP exposure and SSD, but we also revealed a more detailed relationship between DEHP exposure and SSD. Third, to the best of our knowledge, this study is the first to emphasize the interaction between DEHP exposure and DDIF on SSD.

Limitations of this study should also be noted. First, as a part of participants who completed the screening of depression did not undergo urinary DEHP metabolites tests, selection bias might exist. Second, only four kinds of urinary DEHP metabolites were detected, while the other four kinds of urinary DEHP metabolites remained unknown, which might lead to the inaccuracy of the final DEHP concentration. Third, given that the level of urinary DEHP concentration at one time point cannot represent the long-term level of DEHP exposure, it is necessary to test the urinary DEHP concentration at different time points. In addition, a follow-up study on the progress of SSD needs to be supplemented in the future.

## Conclusion

Our study suggests that a higher concentration of DEHP in urine was associated with a higher risk of SSD (<7 h, <6 h, and <5 h). DEHP exposure was found to be associated with SSD <7 h in a sex-specific manner. The ameliorative effects of the median level of DDIR on SSD <7 h in the presence of DEHP exposure was observed in this study. Overall, it is essential to prevent DEHP exposure in daily life.

## Data Availability Statement

Publicly available datasets were analyzed in this study. This data can be found here: https://www.cdc.gov/nchs/nhanes/.

## Ethics Statement

The NHANES Concept was approved by the NCHS Research Ethics Review Board (ERB). Written informed consent was obtained from all individuals or their guardians. The study design was approved by the Ethics Committee of Shanghai Children's Medical Center, School of Medicine, Shanghai Jiao Tong University and conducted according to the Declaration of Helsinki Guidelines (SCMCIRB-W2021065). The patients/participants provided their written informed consent to participate in this study.

## Author Contributions

JL, SC, and JZ conceptualized and designed the study, supervised data collection, carried out the initial analyses, and drafted the initial manuscript. LZhan, JW, and JC coordinated and supervised data collection assisted in the statistical analysis and carried out the initial analyses. MT and SY coordinated and supervised data collection and critically reviewed the manuscript for important intellectual content. LZhao and YY conceptualized and designed the study, supervised data collection, and reviewed and revised the manuscript. All authors read and approved the final manuscript.

## Funding

This work was supported by the Health and Family Planning Scientific Research Project of Pudong New Area Health Committee (PW2021E-06).

## Conflict of Interest

The authors declare that the research was conducted in the absence of any commercial or financial relationships that could be construed as a potential conflict of interest.

## Publisher's Note

All claims expressed in this article are solely those of the authors and do not necessarily represent those of their affiliated organizations, or those of the publisher, the editors and the reviewers. Any product that may be evaluated in this article, or claim that may be made by its manufacturer, is not guaranteed or endorsed by the publisher.

## Abbreviations

SSD: short sleep duration
DEHP: di(2-ethylhexyl) phthalate
ln(DEHP/UCr): ln-transformed urinary creatinine-corrected di(2-ethylhexyl) phthalate
DDIF: daily dietary intake of fiber
OR: odds ratio
CIs: confidence intervals
NHANES: National Health and Nutrition Examination Survey.
