# Supplementary material for: The Relationship Between Daily Dietary Intake of Fiber and Short Sleep Duration in the Presence of Di(2-Ethylhexyl) Phthalate: A Population-Based Study
Source: Front Nutr. 2022 Jun 15;9:910892. doi: 10.3389/fnut.2022.910892 (PMC9240702; doi:10.3389/fnut.2022.910892)
Supplement: Supplementary file 1 [file Data_Sheet_1.PDF]

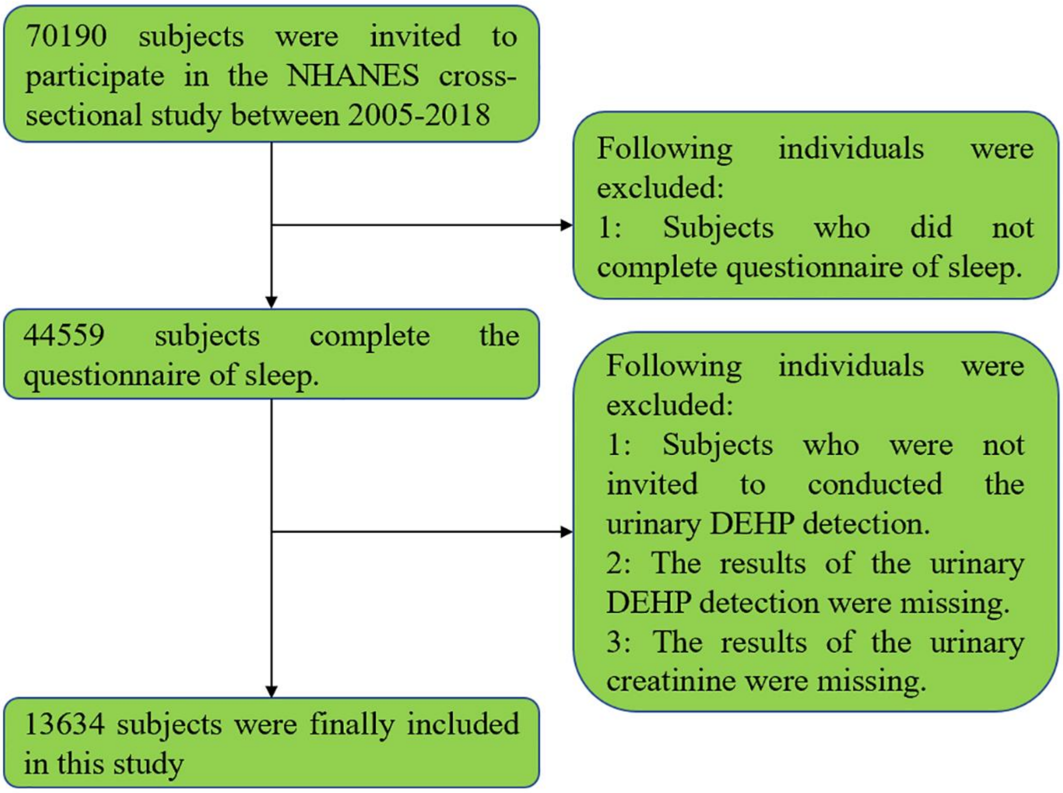

Figure S1: The study flow chart of identifying eligible subjects.

Table S1: Subgroup analyses on the association between DEHP in urine and SSD (<6h)

Subgroup variable	Total number=6383, SSD(<6h)=833		
	$OR_{quartile\ 4\ vs.\ 1}$ (95% CI)	P	$P_{interaction}$
**Sex**			
woman	1.47 (1.17, 1.85)	0.001	
man	1.26 (1.01, 1.57)	0.042	0.345
**Age**			
<60	1.33 (1.10, 1.60)	0.003	
>=60	1.28 (0.95, 1.72)	<0.001	0.953
**Race**			
Mexican American	1.35 (0.87, 2.13)	0.193	
Other Hispanic	0.87 (0.54, 1.40)	0.557	0.116
Non-Hispanic White	1.86 (1.39, 2.49)	<0.001	0.411
Non-Hispanic Black	1.37 (1.04, 1.80)	0.025	0.963
Other Race	0.90 (0.55, 1.45)	0.676	0.199
**Education**			
Below high school	1.26 (0.93, 1.71)	0.145	
High school	1.77 (1.29, 2.46)	0.001	0.096
Above high school	1.24 (0.99, 1.55)	0.066	0.703
**History of diabetes**			

No	1.36 (1.15, 1.62)	<0.001	
Yes	1.26 (0.84, 1.92)	0.272	0.934
**Alcoholic drinks**			
No	1.52 (1.15, 2.02)	0.004	
Yes	1.31 (1.06, 1.61)	0.011	0.360
**History of smoke**			
No	1.34 (1.07, 1.68)	0.010	
Yes	1.37 (1.09, 1.71)	0.006	0.628
**History of hypertension**			
No	1.32 (1.08, 1.61)	0.006	
Yes	1.42 (1.10, 1.85)	0.008	0.565
**DIDF**			
Q1	1.50 (1.18, 1.92)	0.001	
Q2	1.28 (0.98, 1.69)	0.074	0.138
Q3	1.28 (0.96, 1.73)	0.098	0.281

DEHP=MECPP+MEHHP+ MEHP+MEHP; the values of DEHP: Urinary DEHP (ng/ml)/ urinary creatinine (mg/ml); SSD: short sleep duration; DIDF: Daily intake of dietary fiber (g/1000kcal); OR is adjusted for gender (man or woman), age (years old), race(Mexican American, other Hispanic, Non-Hispanic White, Non-Hispanic Black, and Other Race), education (below high school, high school, above high school), history of diabetes (yes or no), and hypertension(yes or no).

Table S2: Subgroup analyses on the association between DEHP in urine and SSD (<5h)

Subgroup variable	Total number=6383, SSD(<5h)=289		
	$OR_{quartile\ 4\ vs.\ 1}$ (95% CI)	P	$P_{interaction}$
**Sex**			
woman	1.48 (1.02, 2.17)	0.042	
man	1.39 (0.98, 1.96)	0.066	0.911
**Age**			
<60	1.35 (1, 1.84)	0.051	
>=60	1.43 (0.92, 2.26)	0.118	0.944
**Race**			
Mexican American	1.06 (0.5, 2.37)	0.873	
Other Hispanic	0.96 (0.44, 2.09)	0.907	0.811
Non-Hispanic White	2.42 (1.51, 4.01)	<0.001	0.117
Non-Hispanic Black	1.08 (0.7, 1.65)	0.722	0.920
Other Race	1.89 (0.83, 4.36)	0.128	0.329
**Education**			
Below high school	1.27 (0.81, 2.03)	0.323	
High school	1.7 (1.04, 2.82)	0.034	0.282
Above high school	1.38 (0.93, 2.04)	0.111	0.784
**History of diabetes**			

No	1.34 (1.01, 1.78)	0.041	
Yes	1.88 (1.01, 3.63)	0.050	0.319
**Alcoholic drinks**			
No	2.22 (1.42, 3.52)	0.001	
Yes	1.13 (0.8, 1.58)	0.495	0.069
**History of smoke**			
No	1.54 (1.07, 2.25)	0.022	
Yes	1.34 (0.94, 1.91)	0.103	0.977
**History of hypertension**			
No	1.34 (0.96, 1.89)	0.091	
Yes	1.54 (1.05, 2.28)	0.030	0.509
**DIDF**			
Q1	1.33 (0.89, 1.99)	0.162	
Q2	1.26 (0.80, 1.97)	0.315	0.470
Q3	1.93 (1.20, 3.15)	0.007	0.611

DEHP=MECPP+MEHHP+ MEHP+MEHP; the values of DEHP: Urinary DEHP (ng/ml)/ urinary creatinine (mg/ml); SSD: short sleep duration; DIDF: Daily intake of dietary fiber (g/1000kcal), Q1:<5.77g/1000kcal, Q2:5.77-9.04g/1000kcal, Q3:>=9.04g/1000kcal; OR is adjusted for gender (man or woman), age (years old), race(Mexican American, other Hispanic, Non-Hispanic White, Non-Hispanic Black, and Other Race), education (below high school, high school, above high school), history of diabetes (yes or no), and hypertension(yes or no).

Table S3: Association between DEHP exposure and short sleep duration(<6h) in different levels of DIDF

	DIDF	N	Model	Quartile of ln(MBP/UCr), range (median) 5.03~7.42 (7.05)	7.42~8.00 (7.73)	8.00~8.67 (8.29)	8.67~14.55 (9.25)	P_{trend}
SSD (<6h)	Q1	4246	Model1	1.00 (Reference)	1.31 (1.03, 1.67)	1.31 (1.03, 1.66)	1.43 (1.13, 1.81)	0.005
			Model2	1.00 (Reference)	1.35 (1.06, 1.73)	1.32 (1.04, 1.69)	1.50 (1.18, 1.91)	0.002
			Model3	1.00 (Reference)	1.34 (1.05, 1.72)	1.31 (1.02, 1.68)	1.50 (1.18, 1.91)	0.002
			Model4	1.00 (Reference)	1.34 (1.05, 1.71)	1.32 (1.03, 1.68)	1.49 (1.17, 1.9)	0.002
	Q2	4260	Model1	1.00 (Reference)	1.02 (0.79, 1.32)	1.30 (1.01, 1.66)	1.07 (0.83, 1.38)	0.263

		Model2	1.00 (Reference)	1.10 (0.85, 1.42)	1.41 (1.09, 1.81)	1.23 (0.94, 1.61)	0.036
		Model3	1.00 (Reference)	1.08 (0.83, 1.41)	1.38 (1.07, 1.79)	1.24 (0.95, 1.62)	0.033
		Model4	1.00 (Reference)	1.10 (0.85, 1.43)	1.42 (1.10, 1.83)	1.23 (0.94, 1.61)	0.035
Q3	42 43	Model1	1.00 (Reference)	0.86 (0.64, 1.16)	1.04 (0.78, 1.38)	1.19 (0.91, 1.57)	0.096
		Model2	1.00 (Reference)	0.90 (0.67, 1.21)	1.10 (0.83, 1.48)	1.29 (0.97, 1.72)	0.030
		Model3	1.00 (Reference)	0.89 (0.66, 1.20)	1.13 (0.85, 1.52)	1.30 (0.97, 1.73)	0.025
		Model4	1.00 (Reference)	0.90 (0.67, 1.21)	1.11 (0.83, 1.49)	1.27 (0.96, 1.70)	0.038

DEHP=MECPP+MEHHP+ MEHP+MEHP; the values of DEHP: Urinary DEHP (ng/ml)/ urinary creatinine (mg/ml); SSD: short sleep duration; DIDF: Daily intake of dietary fiber (g/1000kcal), Q1:<5.77g/1000kcal, Q2:5.77-9.04g/1000kcal, Q3:>=9.04g/1000kcal;

Model1: Unadjusted model

Model2: Adjusted for gender (man or woman), age (years old), race(mexican American, other Hispanic, Non-Hispanic White, Non-Hispanic Black, and Other Race), education (below high school, high school, above high school)

Model3: Adjusted for gender (man or woman), age (years old), race(Mexican American, other Hispanic, Non-Hispanic White, Non-Hispanic Black, and Other Race), education (below high school, high school, above high school), BMI (kg/m2), history of diabetes (yes or no), and hypertension(yes or no).

Model4: Adjusted for gender (man or woman), age (years old), race(Mexican American, other Hispanic, Non-Hispanic White, Non-Hispanic Black, and Other Race), education (below high school, high school, above high school), history of diabetes (yes or no), and hypertension(yes or no).

Table S4: Association between DEHP exposure and short sleep duration(<5h) in different levels of DIDF

	DIDF	N	Model	Quartile of ln(DEHP/UCr), range (median)				P_{trend}
				5.03~7.42 (7.05)	7.42~8.00 (7.73)	8.00~8.67 (8.29)	8.67~14.55 (9.25)	
SSD (<5h)	Q1	4246	Model1	1.00 (Reference)	1.57 (1.08, 2.31)	1.65 (1.14, 2.42)	1.36 (0.93, 2.01)	0.132
			Model2	1.00 (Reference)	1.58 (1.08, 2.32)	1.60 (1.09, 2.35)	1.40 (0.95, 2.09)	0.116
			Model3	1.00 (Reference)	1.54 (1.05, 2.28)	1.58 (1.07, 2.33)	1.42 (0.96, 2.11)	0.107
			Model4	1.00 (Reference)	1.54 (1.05, 2.27)	1.57 (1.07, 2.32)	1.40 (0.94, 2.08)	0.118
	Q2	4260	Model1	1.00 (Reference)	1.22 (0.81, 1.84)	1.09 (0.71, 1.66)	1.14 (0.75, 1.74)	0.694

		Model2	1.00 (Reference)	1.27 (0.84, 1.93)	1.15 (0.75, 1.76)	1.23 (0.80, 1.90)	0.466
		Model3	1.00 (Reference)	1.25 (0.82, 1.89)	1.11 (0.72, 1.71)	1.23 (0.80, 1.90)	0.487
		Model4	1.00 (Reference)	1.26 (0.84, 1.92)	1.14 (0.74, 1.75)	1.19 (0.77, 1.84)	0.555
		Model1	1.00 (Reference)	0.97 (0.59, 1.60)	1.24 (0.78, 2.01)	1.69 (1.09, 2.68)	0.008
Q3	42 43	Model2	1.00 (Reference)	1.02 (0.62, 1.69)	1.35 (0.83, 2.20)	1.89 (1.20, 3.03)	0.002
		Model3	1.00 (Reference)	1.03 (0.62, 1.72)	1.42 (0.87, 2.32)	1.94 (1.22, 3.13)	0.002
		Model4	1.00 (Reference)	1.04 (0.63, 1.72)	1.37 (0.85, 2.25)	1.89 (1.19, 3.03)	0.003

DEHP=MECPP+MEHHP+ MEHP+MEHP; the values of DEHP: Urinary DEHP (ng/ml)/ urinary creatinine (mg/ml); SSD: short sleep duration; DIDF: Daily intake of dietary fiber (g/1000kcal), Q1:<5.77g/1000kcal, Q2:5.77-9.04g/1000kcal, Q3:>=9.04g/1000kcal;

Model1: Unadjusted model

Model2: Adjusted for gender (man or woman), age (years old), race(Mexican American, other Hispanic, Non-Hispanic White, Non-Hispanic Black, and Other Race), education (below high school, high school, above high school)

Model3: Adjusted for gender (man or woman), age (years old), race(Mexican American, other Hispanic, Non-Hispanic White, Non-Hispanic Black, and Other Race), education (below high school, high school, above high school), BMI (kg/m2), history of diabetes (yes or no), and hypertension(yes or no).

Model4: Adjusted for gender (man or woman), age (years old), race(mexican American, other Hispanic, Non-Hispanic White, Non-Hispanic Black, and Other Race), education (below high school, high school, above high school), history of diabetes (yes or no), and hypertension(yes or no).
